# Structural Polymorphism of 441-Residue Tau at Single Residue Resolution

**DOI:** 10.1371/journal.pbio.1000034

**Published:** 2009-02-17

**Authors:** Marco D Mukrasch, Stefan Bibow, Jegannath Korukottu, Sadasivam Jeganathan, Jacek Biernat, Christian Griesinger, Eckhard Mandelkow, Markus Zweckstetter

**Affiliations:** 1 Department for Nuclear Magnetic Resonance (NMR)-Based Structural Biology, Max Planck Institute for Biophysical Chemistry, Göttingen, Germany; 2 Max Planck Unit for Structural Molecular Biology, Hamburg, Germany; 3 Deutsche Forschungsgemeinschaft (DFG) Research Center for the Molecular Physiology of the Brain (CMPB), Göttingen, Germany; Brandeis University, United States of America

## Abstract

Alzheimer disease is characterized by abnormal protein deposits in the brain, such as extracellular amyloid plaques and intracellular neurofibrillary tangles. The tangles are made of a protein called tau comprising 441 residues in its longest isoform. Tau belongs to the class of natively unfolded proteins, binds to and stabilizes microtubules, and partially folds into an ordered β-structure during aggregation to Alzheimer paired helical filaments (PHFs). Here we show that it is possible to overcome the size limitations that have traditionally hampered detailed nuclear magnetic resonance (NMR) spectroscopy studies of such large nonglobular proteins. This is achieved using optimal NMR pulse sequences and matching of chemical shifts from smaller segments in a divide and conquer strategy. The methodology reveals that 441-residue tau is highly dynamic in solution with a distinct domain character and an intricate network of transient long-range contacts important for pathogenic aggregation. Moreover, the single-residue view provided by the NMR analysis reveals unique insights into the interaction of tau with microtubules. Our results establish that NMR spectroscopy can provide detailed insight into the structural polymorphism of very large nonglobular proteins.

## Author Summary

The Tau protein, which plays a central role in the progression of Alzheimer disease, is normally expressed in nerve axons, where it stabilizes microtubules (MTs), supports the outgrowth of axons, and modulates the transport of vesicles and organelles along MTs. In Alzheimer disease, Tau becomes excessively phosphorylated, loses its ability to bind to MTs, and aggregates into intracellular abnormal protein deposits. Many efforts have been made over the years to understand Tau structure as a way to understand Tau function and its mechanisms of action, but these efforts have primarily used traditional biochemistry and molecular biology approaches and therefore have addressed structure and function at a relatively primitive level. Here, we show that it is possible to characterize the structure and dynamics of 441-residue Tau at single residue resolution using nuclear magnetic resonance (NMR) spectroscopy. NMR spectroscopy demonstrates that 441-residue Tau is highly dynamic in solution with a distinct domain character and an intricate network of transient long-range contacts important for pathogenic aggregation. Moreover, the single-residue view provided by the NMR analysis reveals unique insights into the interaction of Tau with MTs.

## Abstract 3

Nuclear magnetic resonance spectroscopy reveals the conformations of Tau, the protein that plays a central role in the progression of Alzheimer disease.

## Introduction

Tau protein was originally discovered as a neuronal microtubule-associated protein (MAP) that stabilizes microtubules (MTs) and supports the outgrowth of axons [1,2]. The protein can modulate the transport of vesicles and organelles along MTs, serves as an anchor for enzymes, and regulates the dynamics of MTs [3,4]. In Alzheimer disease (AD), tau becomes excessively phosphorylated, looses its ability to bind to MTs, and aggregates into neurofibrillary tangles that consist of paired helical filaments (PHFs) of tau. Mutations in the tau gene cause tau aggregation and frontotemporal dementia with parkinsonism linked to Chromosome 17 [5,6].

**Figure 1:**
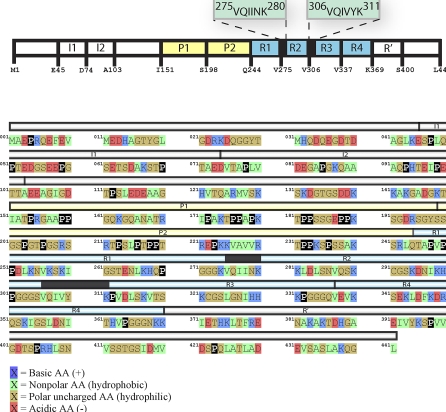
Primary Sequence of 441-Residue Tau (A) Domain organization of htau40. The isoform htau40 is the largest one in the human central nervous system (441 residues). Depending on isoform, the C-terminal half contains three or four pseudorepeats (∼31 residues each, R1–R4, cyan), which are involved in MT binding and form the core of PHFs. The two hexapeptides that are essential for aggregation of tau into PHFs are highlighted. P1 and P2 are the proline rich regions. Domain boundaries are labelled by the residue numbers. (B) Amino acid sequence of htau40. Amino acids are colour coded according to their properties: basic (blue), non-polar (green), polar uncharged (brown), acidic (red). On top of the amino acid sequence, the domain organization is indicated. The two hexapeptides in repeats R2 and R3 are shown as black bars.

The human central nervous system contains six isoforms of tau, generated from a single gene by alternative splicing and ranging between 352 to 441 amino acid residues [7,8]. The isoforms differ by two inserts near the N-terminal end and the presence of either four or three imperfect repeat sequences in the C-terminal half of the protein (Figure 1A). The repeat domain represents the core of the MT interaction [9] and also forms the core of PHFs [10]. For PHF aggregation two hexapeptides at the beginning of the second and third repeats (^275^VQIINK^280^ and ^306^VQIVYK^311^) are crucial because they are able to initiate the aggregation process [11].

It was recognized early on that tau has an unusual character as a protein, because it was resistant to heat and acid treatment without loosing its function and had a very low content of secondary structure [12]. These properties can be traced back to the high fraction of basic and hydrophilic amino acid residues (Figure 1B), which resist the compact folding typical of most proteins. In fact, a number of biophysical studies revealed that tau is a prototypical “natively unfolded” protein [13]. In recent years, this type of protein emerged as a major fraction in the human proteome (termed “natively unfolded” or “intrinsically unstructured proteins” [IUPs] [14]). Apart from tau, most “fibrous” MAPs have the signature of the natively unfolded state, whereas other MT-binding proteins show conventional folding (e.g., motor proteins).

Since disordered proteins tend to be highly flexible and have variable conformations, they have not been amenable for structure analysis by crystallography. Thus nuclear magnetic resonance (NMR) spectroscopy is the only method that allows a description of their conformations and dynamics with high resolution [15]. The lack of an ordered structure, however, causes dramatic signal overlap. Therefore, we and others have previously performed NMR studies on fragments of tau or studied full-length tau but only on the basis of partial assignment that was scattered throughout the sequence [16–25]. In particular by studying tau fragments that contain only the repeat domain (K19 or K18) or the repeat domain and the flanking regions (K32), we and others showed that the hexapeptides in repeats R2 and R3 populate β-structure and bind to MTs and polyanions [16–18], that short stretches in the repeat domain assume highly populated turn conformations [19], that the repeat domain of tau folds into an α-helical conformation upon binding to lipid surfaces [20], and that PHFs formed in vitro by the three-repeat-domain (K19) of tau consist of three major β-strands [21]. Moreover, using a partial assignment of full-length tau (less than 40%) Lippens and coworkers investigated the binding of tau to MTs [22], the phosphorylation pattern of tau as induced by cAMP dependent kinase [23], tau aggregated into PHFs [24], and the impact of binding of heparin to tau [25]. In addition, small angle x-ray scattering (SAXS) and Förster resonance energy transfer (FRET) was used to obtain insight into the structure of the tau protein [26,27].

**Figure 2:**
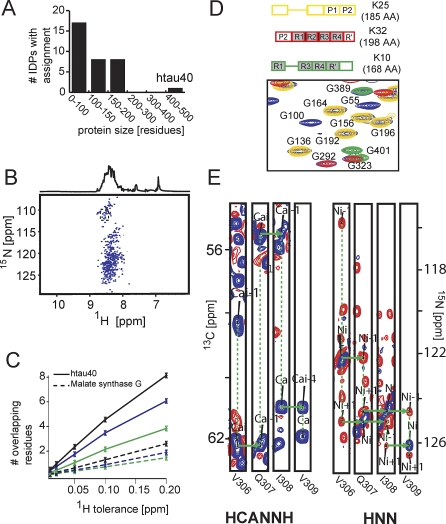
441-Residue Tau at Single-Residue Resolution (A) Number of intrinsically disordered proteins with NMR backbone assignment. (B) ^1^H-^15^N HSQC spectra of htau40 with ^1^H projection on top. (C) Comparison of spectral overlap observed in HSQC spectra of htau40 (solid line) and 731-residue malate synthase G (dashed line). Black, blue, and green indicate ^15^N chemical shift tolerances of 0.2, 0.15, and 0.1 ppm, respectively. (D) Superposition of a selected portion of the ^1^H-^15^N HSQCs of the three tau fragments K25 (yellow), K32 (red), and K10 (green), and of 441-residue htau40 (blue). The domain organization of the three tau constructs is indicated. (E) Assignment strategy for htau40. 2-D strips of high-resolution 3-D HCANNH (left) and HNN spectra (right). The connectivity path linking residues V^306^ to V^309^ is marked in green.

Despite the wealth of information from previous studies on the conformational properties of tau, they were always limited because they were either not of high resolution (SAXS, circular dichcroism, electron microscopy), were restricted to fragments of tau (liquid-state and solid-state NMR, x-ray crystallography of a complexed tau peptide [28]), or were limited to a subset of residues (NMR, FRET). In contrast, we show here that it is possible to obtain the complete backbone assignment of 441-residue tau (the longest tau isoform found in the human central nervous system, htau40; Figure 1B) and thus to overcome the size limitation that in the past has limited detailed NMR studies of unfolded proteins to fewer than 200 amino acids (Figure 2A) [16,29]. The complete backbone assignment of htau40 allowed us to probe the structure and dynamics of the full-length soluble protein, including the 198 residues of the N-terminal half and the 47 residues of the C-terminal domain, and determine at single residue-resolution the residues involved in the interaction between tau and MTs. Most importantly, the data provide unique insights into long-range interactions between remote regions of tau that can be studied only in the context of the full-length protein.

## Results

### Backbone Resonance Assignment of 441-Residue Tau

For 441-residue htau40, we observed a narrow, highly congested cluster of amide proton signals in a 1-D NMR spectrum (Figure 2B). Correlation with the directly attached ^15^N-amides in a two-dimensional^ 1^H-^15^N heteronuclear single quantum coherence (HSQC) spectrum, only partially resolved the degeneracy (Figure 2B): the number of overlapping signals was still a factor of 3.5 higher than in the largest currently assigned globular protein, the 731-residue malate synthase G (Figure 2C). The large number of proline residues (43 out of 441 residues) and the strongly repetitive primary sequence in the repeat domain of htau40 also complicated the analysis of sequential connectivity. Within a range of +/−0.2 ppm we observed 20.1 residues on average (Figure S1).

To obtain the sequence-specific assignment of the backbone resonances of htau40, we recorded 3-D (HA)CANNH [30] and HNN [31] experiments. For nitrogen and C^α^ nuclei, very high resolution was obtained at the second highest currently commercially available magnetic field (21.14 T) (Figure 2E), and more than 98% of non-proline backbone resonances for the full length htau40 protein were assigned. Thus, htau40 exceeds more than 2-fold the largest currently assigned disordered protein. Only Gly^272^, Gly^303^, Gly^334^, and Gly^366^, which are found at the C-terminal end of each repeat region and are surrounded by two glycines in the sequence motif PGGG, as well as Gly^304^ and Gly^335^ could not be assigned unambiguously owing to severe signal overlap. In addition, the resonances originating from Met^1^ and Ala^2^ were not observed in ^1^H-^15^N HSQC spectra. In case of proline, more than 83% of C^α^ frequencies were assigned. The assignment was corroborated by producing three overlapping fragments (Figure 2D): (i) a 185-residue fragment comprising the N-terminal half up to the repeat region, but excluding the two inserts that are affected by alternative splicing (I1, I2, encoded by exons 2 and 3); (ii) a 198-residue fragment containing the repeat region and the two proline-rich flanking regions; (iii) a 168-residue fragment covering most of the C-terminal half except for the second repeat R2. The two 29-residue inserts in the N-terminal half were only present in htau40. Superposition of HSQCs of the three fragments with that of htau40 showed that many resonances observed in the three fragments were found at identical positions as in htau40 (Figure 2D), in agreement with its high flexibility. This dataset forms the basis for probing intramolecular interactions and studying the interactions between tau and its binding partners.

### Secondary Structure Propensity in 441-Residue Tau

**Figure 3:**
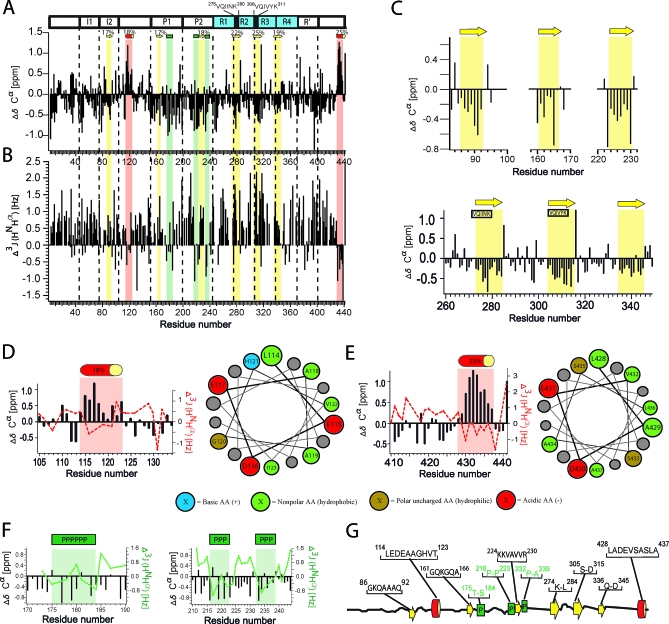
Residual Secondary Structure of Tau in Solution (A) Secondary chemical shifts for C^α^. Regions of β-structure (α-helical) propensity are identified by negative (positive) values extending over several residues and are highlighted in yellow (red). Regions that preferentially populate polyproline II helix have negative C^α^ secondary chemical shifts and are marked in green. The domain organization of htau40 is shown above. Repeat boundaries are indicated by vertical dashed lines. (B) Differences between experimental ^3^J(H^N^H^α^) scalar couplings and random coil values as a function of sequence number. Regions of β-structure (α-helical) propensity are identified by positive (negative) values extending over several residues. Regions that preferentially populate polyproline II helix have negative Δ^3^J(H^N^H^α^) values and can therefore readily be distinguished from β-structure. (C) C^α^ secondary chemical shifts of regions with a propensity to adopt β-structure. (D,E) Comparison of C^α^ secondary chemical shifts with Δ^3^J(H^N^H^α^) values in regions of transient helical structure. On top, the estimated population of α-helical structure is indicated. Mapping onto a helical wheel reveals two amphiphatic helices. (F) Comparison of C^α^ secondary chemical shifts with Δ^3^J(H^N^H^α^) values in regions that transiently populate polyproline II helical conformations. (G) Schematic representation of the elements of transient secondary structure in htau40: β-structure (yellow), α-helical (red), polyproline II (green). In case of β-structure propensity, only regions with populations of 17% or more are shown.

NMR spectroscopy provides a variety of probes that are highly sensitive to backbone dihedral angles in both globular and disordered proteins [32]. We used experimental C^α^ chemical shifts and ^3^J(H^N^H^α^) couplings, from which random coil values were substracted to reveal the presence of helical or β-structure. For all residues of htau40, C^α^ secondary chemical shifts were below 1.5 ppm (positive or negative) (Figure 3A), indicating that rigid secondary structure elements are not present in htau40. However, several continuous stretches (containing 7–11 residues) with negative C^α^ secondary chemical shifts were observed in the repeat region (Figure 3A and 3C), indicative of a propensity to adopt β-structure. According to a quantitative analysis, the β-structure-like conformations are populated 12%, 22%, 25%, 19%, and 12% of the time for residues ^256^VKSKIG^262^ (in R1), ^274^KVQIINKKLDL^284^ (in R2), ^305^SVQIVYKPVDL^315^ (in R3), ^336^QVEVKSEKLD^345^ (in R4), and ^351^QSKIGSL^357^ (in R4), respectively. In addition, stretches of negative C^α^ secondary chemical shifts consisting of more than five amino acids were found for ^86^GKQAAAQ^92^ (17% in I2), ^161^GQKGQA^166^ (17% in P1), and ^224^KKVAVVR^230^ (18% in P2). Thus, the highest β-structure content was found for residues ^274^KVQIINKKLDL^284^ and ^305^SVQIVYKPVDL^315^ in repeats R2 and R3, comprising the two aggregation-prone hexapeptides ^275^VQIINK^280^ and ^306^VQIVYK^311^. Formation of β-structure in the homologues region of R1 (^243^LQTAVMPDL^253^) is hindered by the presence of three proline residues (Figure 1B).

Continuous stretches of positive secondary chemical shifts report on α-helical propensity and were observed for ^114^LEDEAAGHVT^123^ (between insert 2 [I2] and the proline-rich region P2) and ^428^LADEVSASLA^437^ in immediate proximity to the C terminus (Figure 3A, 3D, and 3E). Quantitative analysis revealed 18% of α-helical population for ^114^LEDEAAGHVT^123^ and 25% for ^428^LADEVSASLA^437^. Mapping of these two residue stretches onto a helical wheel reveals two amphiphatic helices with more hydrophobic residues on one side of the helical cylinder and an excess of negative charges on the opposite side (Figure 3D and 3E).

The ^3^J_NH-αH_-coupling of a residue depends on its φ backbone torsion angle. Positive Δ^3^J_NH-αH_ (J_exp_−J_random coil_) values indicate a tendency towards extended and β-structure, low or even negative values indicate turns or helical propensity. In htau40, positive Δ^3^J_NH-αH_ values dominate along the entire sequence indicating its overall extended chain conformation (Figure 3B). The largest ^3^J_NH-αH_ values were detected for residues ^305^SVQIVYKPVDL^315^, in agreement with the highest β-structure propensity (25%) as estimated from C^α^ secondary chemical shifts.^ 274^KVQIINKKLDL^284^ also showed increased positive Δ^3^J_NH-αH_ values, but the effect was less pronounced. In contrast, negative Δ^3^J_NH-αH_ values or values close to zero were found for ^114^LEDEAAGHVT^123^ and ^428^LADEVSASLA^437^ (Figure 3B, 3D, and 3E), supporting the preferential population of α-helical conformations by these residues. In addition, small ^3^J_NH-αH_-couplings were detected for several non-proline residues in the two proline-rich regions P1 and P2 (Figure 3B and 3F): Thr^175^, Ala^178^, Thr^181^, Ser^184^, Thr^217^, Thr^220^, Glu^222^, Lys^224^, Lys^234^, and Ser^235^. Most of these residues also showed negative or very small positive C^α^ secondary chemical shifts (Figure 3F), suggesting that ^175^TPPAPKTPPS^184^, ^216^PTPPTREP^223^, and ^232^PPKSPSSA^239^ transiently assume polyproline II helical conformations (Figure 3G). Two of these motifs (^216^PTPPTREP^223^ and ^232^PPKSPSSA^239^) are separated by a stretch of positive Δ^3^J_NH-αH_ values (Figure 3F), consistent with the β-structure propensity of ^224^KKVAVVR^230^ that was suggested by C^α^ chemical shifts.

**Figure 4:**
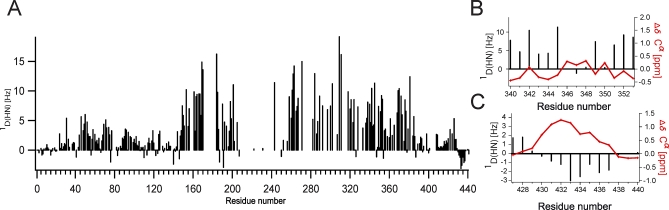
RDCs Observed in Weakly Aligned Tau (A) Profile of ^1^D(HN) dipolar couplings observed in htau40 aligned in Pf1 bacteriophage at 5 °C. Increased positive (for the β-structures and polyproline II helix regions) as well as negative (for the α-helices) RDCs indicate rigidity on the nano- to microsecond time scale. (B,C) Comparison of ^1^D(HN) dipolar couplings (black bars) with C^α^ secondary chemical shifts (red line) in the turn region ^345^DKFD^348^ (B) and the helical region ^428^ladevsasla^437^ (C). Sign-inversion of RDCs indicates that the H-N internuclear vectors are parallel to the long axis of the corresponding segment.

Residual dipolar couplings (RDCs) [33] report on time and ensemble-averaged conformations [34] and can be used to understand both the structure and dynamics of disordered proteins [35]. By weakly aligning htau40 in Pf1 bacteriophage, we could determine 262 residual one-bond H-N dipolar couplings (Figure 4). For other residues, peak overlap prohibited a quantitative analysis. In addition, most residues in the proline-rich region P2 (residues 198–244) as well as residues 171–183 in the proline-rich region P1 showed very strong alignment prohibiting a quantitative analysis of their dipolar couplings. Large residual one-bond H-N dipolar couplings were observed in the repeat domain and the proline-rich regions P1 and P2. In the repeat region, the largest values were found for the hexapeptide ^306^VQIVYK^311^ in the beginning of R3 (Figure 4A). Large positive H-N RDCs are associated with locally more extended conformations [35], in qualitative agreement with β-structure propensity of ^306^VQIVYK^311^.

**Figure 5:**
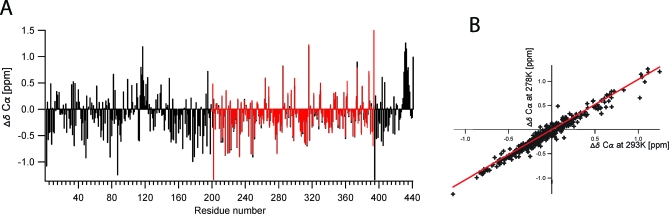
Robustness of Secondary Structure Propensity of Tau (A) Superposition of C^α^ secondary chemicals shifts observed in 441-residue Tau (black) and in a 198-reside fragment (K32) comprising only the repeat domain and its flanking regions (shown as red bars). (B) Correlation of C^α^ secondary chemicals shifts observed in htau40 at 278K and 293K.

Negative H-N RDCs were observed for 43 residues (Figure 4A). In particular, residues 430–438 showed negative RDC values, with the most negative value (−12.4 Hz) found for S433 (Figure 4A and 4C). The sign inverted RDCs indicate that the H-N internuclear vectors of ^428^LADEVSASLA^437^ are parallel to the long axis of this segment [36], in agreement with the presence of a helical conformation. Isolated residues with sign-inverted RDCs are characteristic for the presence of turns in disordered proteins [19]. In htau40, most of the negative H-N RDCs belong to residues in the N-terminal part (residues 1–200), suggesting a higher number of turns in this region. Previously, we showed by a combination of H-N RDCs and molecular dynamics simulation that the four peptides ^252^DLKN^255^, ^283^DLSN^286^, ^314^DLSK^317^, and ^345^DKFD^348^, embedded in a fragment that only comprised the repeat domain of tau (K18), showed high propensities to form turns [19]. In htau40, the peak overlap was strongly increased (Figure 2), and we could analyze reliably only K^347^, which showed a H-N RDC value of −4.6 Hz (Figure 4B). However, the C^α^ secondary chemical shifts observed for ^252^DLKN^255^, ^283^DLSN^286^, ^314^DLSK^317^ in K32 and htau40 were nearly identical (Figure 5A), suggesting that the four peptides also populate turn conformations in htau40.

### Temperature and Construct Dependence of Secondary Chemical Shifts

Comparison of the H-N HSQC spectra showed that most cross peaks of the three fragments (K25, K32, and K10; Figure 1) were found in very similar positions as in the htau40 spectrum. To further probe the influence of flanking domains on the structural propensities of different regions of htau40, we compared secondary chemical shifts of the three fragments with values observed in full-length tau. Close agreement was found between C^α^ secondary chemical shifts of K25, K32, K10, and htau40 (Figure 5A and S2). The largest deviation between any of these fragments and htau40 was 0.32 ppm. The rmsd values were 0.04, 0.05, 0.04 ppm for the comparisons K32-htau40 (171 residues), K25-htau40 (168 residues), and K10-htau40 (150 residues), respectively.

To further probe the robustness of the local conformational properties of htau40, we determined the sequence-specific assignment of backbone resonances at 20 °C. Backbone resonance assignment at 20 °C was achieved by following the shifts of cross peaks in H-N HSQCs of htau40 supported by 3D (HA)CANNH spectra of K25, K32, K10, and htau40 at 20 °C. The C^α^ secondary chemical shifts observed at 20 °C in htau40 were highly similar to the values observed at 5 °C (Figure 5B). We conclude that the structural propensities of monomeric tau are highly specific conformational fingerprints.

### Flexibility of the Backbone of htau40

**Figure 6:**
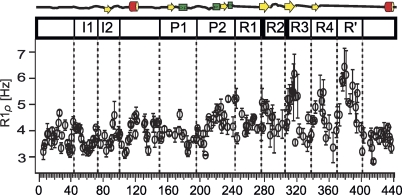
Intrinsic Flexibility of Tau Plot of ^15^N R_1ρ_ spin relaxation rates along the amino acid sequence. High R_1ρ_ rates reflect increased rigidity on a pico- to nanosecond time scale and are mostly found in regions that transiently populate extended structures (β-structure or polyproline II helix).

To probe the dynamics of the backbone of htau40, we measured spin relaxation rates. ^15^N R_1ρ_ spin relaxation rates allow quantification of motions that occur on timescales of pico- to nanoseconds and micro- to milliseconds and reflect the flexibility of the protein in solution [37]. For the N-terminal domain up to residue 170, R_1ρ_ rates were below 4.7 Hz with an average value of 3.8 Hz (Figure 6), indicating that this part of tau is highly mobile. In the proline-rich region P2, the R_1ρ_ values increased and reached a maximum of 5.2 Hz for S^235^, indicating increased rigidity. The observed maximum is part of the ^232^Pro-Ala^239^ stretch that transiently populates polyproline II helical conformations, suggesting this forms a more periodic and less flexible structure. Similarly, we also observed R_1ρ_ spin relaxation rates above 4.7 Hz for many residues that belong to elements of transient β-structure in the repeat domain (see above): Ile^277^, Asn^279^, and Leu^282^ (in R2), ^309^Val-Lys^321^ (in R3), and ^343^Lys-Lys^353^ (in R4). The largest R_1ρ_ rates were detected for ^370^Lys-Lys^395^ in the region downstream of the repeat domain.

RDCs are not only excellent probes for structure, but are also sensitive to motions from picoseconds to milliseconds [38]. The large H-N RDC values, which were observed in the repeat domain (Figure 4), arise from locally more extended conformations that at the same time increase the rigidity of this region. Interestingly, R_1ρ_ relaxation rates are not elevated in the proline-rich domain P1, whereas H-N RDCs in this region are a factor of two or more larger than for residues 1–140. This suggests that proline residues restrict the mobility of the backbone in the time window between the global correlation time of the protein and 50 μs that is invisible to NMR relaxation measurements.

### Global Folding of Soluble Tau

**Figure 7:**
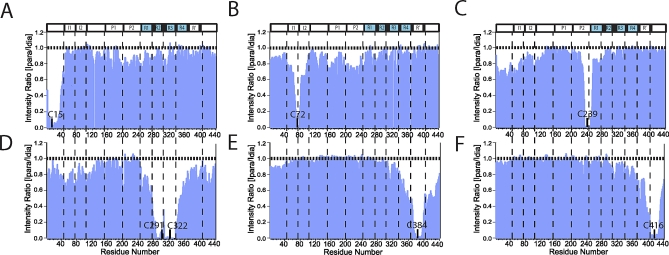
PRE of Amide Protons in Spin-Labelled Tau (A–F) PRE profiles of amide protons in spin-labelled htau40. *wt* htau40 and five single-cysteine mutants (A15C, A72C, A239C, A384C, and A416C) were labelled with MTSL at residue (A) 15, (B) 72, (C) 239, (D) 291 and 322, (E) 384, and (F) 416. Intensity ratios were averaged over a three-residue window. Decreases in peak intensity ratios that occur far from the site of spin-labelling (>10 residues) are indicative of long-range contacts (<25 Å) between the spin-label and distant areas of sequence.

To study the global folding of htau40, we employed paramagnetic relaxation enhancement (PRE) of NMR signals [39]. The primary sequence of htau40 contains two cysteines (C291 and C322) that provide convenient attachment points for the nitroxide radical (1-oxy-2,2,5,5-tetramethyl-D-pyrroline-3-methyl)-methanethiosulfonate (MTSL). In addition, five different mutants, which harbour only a single cysteine in the projection domain (A15C or A72C), in the proline-rich region (A239C), or near the C terminus (A384C or A416C), were constructed and labelled with MTSL. Figure 7 shows the PRE profile (ratio of NMR signal intensities in the paramagnetic and diamagnetic state) of the amide protons of htau40 for the six different MTSL-labelled htau40 samples. For a fully extended chain, the NMR signal intensities in the paramagnetic and diamagnetic state should be identical for residues that are more than ten to 15 residues away from the position of the spin label. Thus, if htau40 would be a fully extended chain most residues will show a PRE intensity ratio of one. In clear contrast, we observed for many residues far from the site of spin-labelling PRE intensity ratios below one, indicative of transient long-range contacts between the spin-label and distant areas of sequence (Figure 7). When the spin label was attached to position 15, intensity ratios of approximately 0.8 were observed in the proline-rich domain P2 (residues 200–240) as well as residues downstream of P2 (residues 130–200) and residues in the repeat region up to residue 330 (Figure 7A). A transient long-range interaction between the N-terminal region and its central domain is further supported by the PRE profile of C239-MTSL labelled htau40, for which intensity ratios of 0.6–0.8 were observed for residues 1–80 and weaker broadening extended up to residue 150 (Figure 7C). On the other hand, signal intensity ratios in case of C291/C322- and C384-MTSL labelled htau40 indicate that the C-terminal domain (residues 360–441) transiently contacts the repeat region and the 40 N-terminal residues (Figure 7D and 7E).

### Ensemble of Structures Populated by htau40 in Solution

To obtain more direct insight into the ensemble of structures populated by htau40 in solution, we converted all NMR signal intensity ratios <0.9 into distance restraints using the *r* ^−6^ dependence of the PRE effect on the electron–proton distance [39]. In this way we obtained—from the six PRE profiles shown in Figure 3—1,224 intramolecular long-range contacts ranging between 0 Å and 25 Å. In addition, PRE intensity ratios close to one (here >0.9) indicate that the corresponding amide proton is on average more than 25 Å away from the spin label, allowing lower distance boundaries of 25 Å for these residues. The total of 2,288 PRE distance restraints was subjected to a structure calculation using simulated annealing [40]. Structure calculations of proteins are challenging when the protein exchanges rapidly between different conformations, such that only a single NMR signal is observed. Rapid exchange between multiple conformations is clearly present in the highly dynamic tau protein and the PRE intensity ratios shown in Figure 7 are values averaged over a large ensemble of conformations. Moreover, due to the *r* ^−6^ dependence, conformations with short intramolecular distances contribute more strongly to the PRE broadening than more extended structures. To take into account the dynamic nature of htau40 we performed both single molecule calculations, in which all distance restraints were enforced simultaneously onto a single molecule, as well as ensemble calculations in which the PRE distance restraints had to be fulfilled not by single structure but collectively by an ensemble of 30 conformations, respectively [41,42]. Clearly, even the 30 conformer calculations are a compromise and underestimate the number of conformations htau40 can assume in solution. Nevertheless they better reflect the ensemble nature of the PRE distances, i.e., the observed PRE broadening arises from a very large number of different conformations and each conformation only fulfils a small subset of distance restraints at any given time. On the other hand, single molecule calculations can allow direct access to the more compact conformations that htau40 could potentially assume in solution.

**Figure 8:**
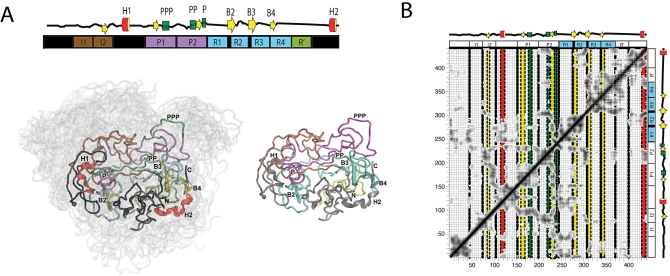
Native-State Conformations of 441-Residue Tau in Solution (A) Representation of the conformations of htau40 calculated from PRE data. Left panel: Colour coding follows the domain organization diagram shown above. Regions of transient α-helical structure (H1[114–123] and H2[428–437]) and β-structure (B2[274–284], B3[305–315] and B4[336–345]) are shown in red and yellow, respectively. Polyproline II stretches (PPP[175–184], PP[216–223], and P[232–239]) are coloured green. In the background, an ensemble of 20 conformations is shown. Right panel: Same conformation as in left panel, but colour coding according to the domain organization of tau. (B) Average contact map for the seven lowest-energy structures obtained from a calculation in which all distance restraints were enforced onto a single molecule. A continuous grey scale from 3 Å (black) to 22 Å (white) is used.

The structure calculations in which all distance restraints were enforced onto a single molecule resulted in an ensemble of compact conformations (Figure 8A). The shown structures fulfil all 2,288 experimental distance constraints within 0.5 Å. It is readily apparent that many different conformations are in agreement with the experimental PRE distance restraints (structures shown in light grey). The conformation highlighted as ribbon diagram in Figure 8A has a radius of gyration *R*_g_ of 48 Å and is therefore at the lower end of the distribution of R_g_ values obtained from SAXS [26]. Subsequently, this conformation was used in the ensemble calculations. In agreement with the fact that a single compact structure could fulfil all distance restraints, the same was true for structures calculated by ensemble averaging. However, as distance restraints only had to be fulfilled by an ensemble of structures, more expanded conformations were obtained. The average radius of gyration of the ensemble of calculated structures was approximately 65 Å, in agreement with the average value obtained for htau40 from SAXS [26].

htau40 is a highly dynamic protein and many conformations are in agreement with the experimental PRE profiles (Figure 8A). To extract long-range interactions that occur in many of the calculated structures, we determined all C^α^ distances in each structure and averaged the resulting contact map over the ensemble of structures (Figure 8B). Thus, dark spots in the contact map shown in Figure 8B indicate conserved intramolecular interactions. In detail, the following structural properties of htau40 were revealed: (i) The N-terminal 50 residues favour a compact conformation, as indicated by strong contacts within the residue stretch 1–20 and from this region to residues 30–50 (lower left corner in Figure 8B). (ii) The N-terminal 50 residues contact the C-terminal domain, as indicated by the contacts between residues 1–50 and residues 380–400 and seen in the PRE profile of C384-MTSL htau40 (Figure 7E). (iii) Inserts I1 and I2 fold back onto each other, as indicated by the short antidiagonal crossing the diagonal of the contact map at approximately the boundary between I1 and I2. (iv) Residues 113–124 interact with the N-terminal end of I1 (residues E45–D74). (v) The region separating I2 from the proline-rich region has a high propensity for compaction. (vi) Large regions of the N-terminal domain interact with the proline-rich region P2 and repeats R1 to R3. (vii) Residue stretches in the proline-rich domains, which transiently assume polyproline II helical conformations, are in contact. (viii) The proline-rich regions P1 and P2 interact with the hexapeptide in repeat R3. (iv) Repeats R1 and R2 assume compact conformations, favoured by the presence of turns in this region [19]. (v) Repeats R3 and R4 contact the C-terminal domain.

### Dependence of Long-Range Contacts on Ionic Strength and Urea

**Figure 9:**
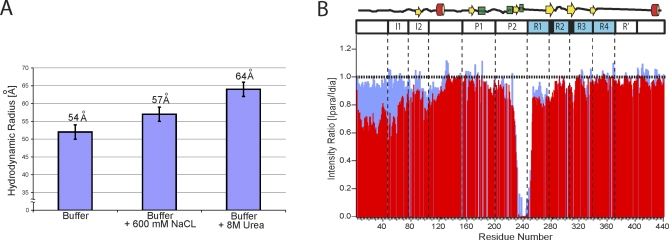
Influence of Ionic Strength and Urea on Intramolecular Long-Range Interactions (A) Hydrodynamic radius values of htau40 in buffer (99.9 % D_2_O, 50 mM phosphate buffer [pH 6.9]), upon addition of 600 mM NaCl and in the presence of 8 M urea. (B) PRE profiles of amide protons in spin-labelled htau40 in buffer (red bars) and in the presence of 600 mM NaCl (blue bars). The single-cysteine mutant A239C was labelled with MTSL. Intensity ratios were averaged over a three-residue window.

To obtain insight into the nature of the long-range interactions observed in htau40, we performed NMR diffusion measurements in 50 mM phosphate buffer as well as in the presence of 600 mM NaCl and 8 M urea. NMR diffusion experiments allow estimation of the hydrodynamic radius of a protein in solution and therefore allow a global assessment of intramolecular long-range interactions [43]. For htau40 in buffer, the diffusion properties indicate a hydrodynamic radius of 54 Å (Figure 9A). Taking into account that for natively unfolded proteins the radius of gyration (*R*_g_) is about 1.2 to 1.5 times larger than the hydrodynamic radius [44], this is consistent with an *R*_g_ value of 65 Å of htau40 as determined by SAXS [26]. In the presence of 600 mM NaCl the hydrodynamic radius of htau40 was increased to 57 Å, and in the presence of 8 M urea further to 64 Å (Figure 9A). In agreement with the increased hydrodynamic radius values, PRE-broadening between the spin label attached to residue 239 and the N-terminal domain was strongly reduced when the ionic strength was raised to 600 mM NaCl (Figure 9B).

### Interaction of Tau with MTs

**Figure 10:**
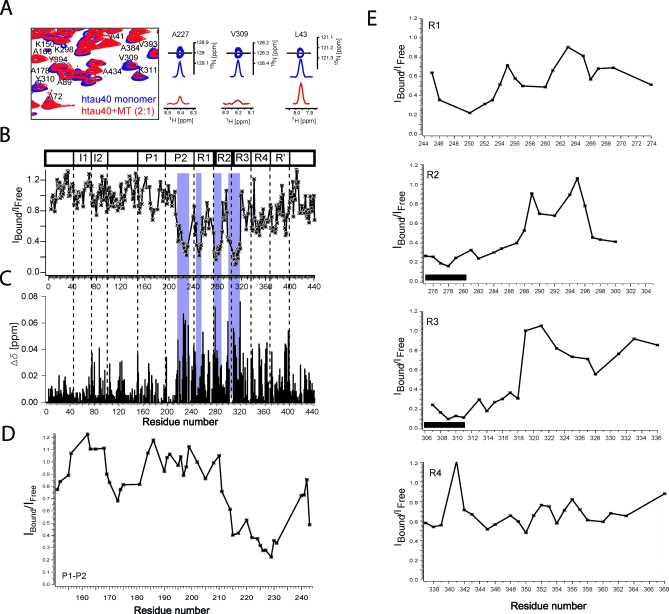
Interaction of Tau with MTs (A) Superposition of a selected portion of the ^1^H-^15^N HSQC spectra of htau40 in the free state (blue) and in the presence of MTs (red). Resolved peaks are labelled with their assignments. The tau:tubulin heterodimer ratio was 2:1. To highlight changes in NMR signal intensities, a trace through the center (solid line) of selected peaks is shown. (B) NMR signal intensity ratios between signals observed for htau40 in the MT-bound and in the free state. Repeat boundaries are indicated by vertical dashed lines. Hot spots of binding are highlighted in blue. (C) Absolute magnitude of ^15^N chemical shift changes. The estimated error based on ^1^H-^15^N HSQCs of two different htau40 samples was 0.025 ppm. (D,E) NMR signals intensity ratios in the proline-rich regions (D) and in the repeat domain (E). Values were taken from (B). In (E), the position of the two hexapeptides is indicated by black bars.

The binding of htau40 to MTs was characterized using NMR chemical shift perturbation in 2-D ^1^H-^15^N HSQC spectra. As shown previously, taxol-stabilized MTs are stable at 5 °C for several hours, sufficient for the time course of the NMR experiment [16]. Upon addition of taxol stabilized MTs to monomeric htau40 a nonuniform reduction of signal intensities in a ^1^H-^15^N HSQC of htau40 was observed (Figure 10A and 10B). The broadening is caused by an exchange of tau molecules between the free and the MT-bound state that is intermediate on the NMR time scale. Strong signal broadening was observed for residues in the proline-rich region P2 and in repeats R1 to R3. For residues 214 to 241 in P2, signal intensities were reduced to below 70%. Within this region two minima were present, comprising residues Leu^215^ and ^225^KVAVVRT^231^ (Figure 10D). In the unbound state, ^224^KKVAVVR^230^ preferentially populate β-structure, whereas the two neighbouring residue stretches (^216^PTPPTREP^223^ and ^232^PPKSPSSA^239^) have a propensity for polyproline II helix (Figure 3G). In P1, ^170^RIPAKTPPAPKT^181^ showed a more pronounced broadening than other residues (Figure 10D). Interestingly, part of this stretch preferentially populates polyproline II helical conformations in the free state (Figure 3). In the repeat domain, strong signal broadening was observed for 13 residues in the beginning of repeats R2 and R3 with the minima located at I278 and V309 (Figure 10E). In addition, residues in the homologues region of R1 were strongly attenuated in the presence of MTs, although the signal reduction was more restricted and not as pronounced as in R2 and R3. Significantly less chemical exchange broadening was present in the N-terminal parts of R4 and R' (Figure 10B and 10E).

In agreement with the observed chemical exchange broadening, the presence of MTs induced chemical shift changes for residues in P2, R1–R4, and R' (Figure 10C). In addition, ^15^N chemical shift changes exceeding 0.025 ppm were observed for V75, T76, V80, V88, V122, A125, and I151 (Figure 3). As the N-terminal 150 residues of htau40 do not strongly contribute to binding and assembly of MTs [22,45], the chemical shift changes might be attributed to weak transient contacts with MTs or to changes—as a result of MT binding—in intramolecular long-range interactions in htau40.

## Discussion

### Intrinsically Disordered Tau

Tau is important for neuronal cell biology because it stabilizes MTs and promotes axonal outgrowth, and for neurodegeneration because it undergoes abnormal aggregation in AD and other brain disorders [3,5,6]. However, the mode of action of tau is still enigmatic. As soon as the protein was discovered [1], its unusual behaviour became apparent because it retained its function even after heat denaturation. Subsequent biophysical characterization revealed that tau was highly soluble and almost devoid of secondary structure [12]. Cloning of the protein confirmed a high fraction of hydrophilic amino acid residues and an overall basic character, complementary to the acidic surface of MTs [7,8]. It also revealed three or four semiconserved repeats of ∼31 residues that were involved both in the interactions with MTs and in the assembly of Alzheimer PHFs. However, the function of MTs was curiously distributed over many residues, each contributing only weakly [9]. Electron microscopy studies of tau showed that the molecule had very little contrast, and only special surface-rendering methods revealed tau as irregular elongated molecules [46,47]. X-ray scattering, circular dichroism (CD), and Fourier transform infrared (FTIR) studies all pointed to a seemingly random structure in solution that was termed “natively unfolded” [13].

NMR spectroscopy provided now a detailed view of the natively unfolded nature of 441-residue tau at single residue resolution. 343 out of 441 residues of the htau40 monomer are in a nonperiodic, disordered conformation (Figure 3). Transient elements of secondary structure were restricted to small regions (Figure 3G): (i) ^274^Lys-Leu^284^, ^305^Ser-Asp^315^, and ^336^Gln-Asp^345^ transiently populate β-structure, in agreement with previous studies on fragments covering the repeat region [17,18]. The high propensity of β-structure in the parts that are essential for PHF formation (^274^Lys-Leu^284^ and ^305^Ser-Asp^315^) underpins the fact that these residues serve as seeds of aggregation. (ii) ^175^TPPAPKTPPS^184^, ^216^PTPPTREP^223^, and ^232^PPKSPSSA^239^ in the proline-rich regions P1 and P2, transiently assume polyproline II helical conformation. These are the only short residue stretches in htau40 that comprise at least three prolines of which two are sequential (Figure 1B). Within the motifs ^175^TPPAPKTPPS^184^, ^216^PTPPTREP^223^, and ^232^PPKSPSSA^239^ in the proline-rich region, there are several phosphorylation sites that are characteristically elevated in AD, that is, Thr^175^, Thr^181^, Thr^231^, Ser^235^ [48]. Moreover, several antibodies against phosphorylated tau require dual phosphorylation, separated by three to four residues, e.g., 202 + 205, or 231 + 235. When a polyproline II helical structure is formed the two phosphorylated residues would be facing the same way on the helix. However, in the free state of htau40 the site that is recognized by the AT8 antibody (residues ^199^SPGSPGT^205^) does not show a clear propensity to adopt polyproline II helix. (iii) ^114^Leu-Thr^123^ transiently populates α-helical structure. The helical structure might promote intramolecular long-range interactions in tau and might be important for interaction with the dynein-activator complex dynactin [49]. Notably, Thr^123^ is one of only a few residues in the N-terminal domain that is phosphorylated in PHF tau [50]. (iv) ^428^Leu-Ala^437^ have a high propensity to form α-helical structure. Notably, truncation of the C terminus behind Asp^421^ was suggested to be an early molecular event in tau aggregation [51], suggesting that the conformational properties of ^428^Leu-Ala^437^ can influence proteolytic cleavage.

Based on their functional differences three different domains of tau were defined: (i) the projection domain comprising residues 1–200, i.e., the N-terminal part of tau up to the proline-rich region P2, (ii) the central region comprising the repeat domain and its flanking regions P2 and R', and (iii) the 40–50 C-terminal residues [52]. NMR dipolar couplings (Figure 4) demonstrated that the functional differences are associated with strong differences in the intrinsic flexibility of the three domains. Whereas the repeat domain and its flanking proline rich regions have a lower intrinsic flexibility on a time scale in the nanosecond to microsecond range detected by dipolar couplings, which is important for formation of secondary structure, in agreement with an increased propensity to populate polyproline II or β-structure, residues in the projection domain as well as in the C-terminal region more rapidly interconvert between different conformations, which is detected by relaxation measurements. The differences in intrinsic mobility are associated with a decreased number of hydrophobic and increased number of negatively charged residues in the projection domain (Figure 1B). Moreover, using NMR dipolar couplings a reduced mobility in regions that harbour many proline residues had been previously observed in the C-terminal tails of α- and β-synuclein [53,54]. Importantly, Figure 4 is also very suggestive of a possible folding of the N-tail and C-tail over the middle domain of tau, previously termed the paperclip model [27].

### Intricate Network of Long-Range Interactions in Soluble Tau

The appearance of tau as an elongated molecule by some EM methods [46,47] suggested that the conformation in solution was extended in agreement with the natively unfolded state of tau and the accessibility to multiple kinases throughout the chain. However, evidence for global folding began to emerge from several antibodies that had discontinuous epitopes comprising residues near the N terminus and within the repeat domain (e.g., Alz-50, MC-1 [55]). This evidence was further confirmed by FRET studies showing that tau was able to form a double hairpin, leading to a “paperclip” structure whereby both N- and C terminus were folded into the vicinity of the repeat domain [27]. This concept is now substantially expanded and refined by the NMR analysis (Figure 8). Whereas fluorescence resonance energy transfer combined with electron paramagnetic resonance requires two labels, one label acting as donor and the other one as acceptor, PRE monitored by NMR requires only a single paramagnetic centre such as MTSL attached to a free cysteine. Even more important, whereas in FRET only a single distance can be measured for each donor-acceptor pair, all nuclei in the protein serve as acceptor. Thus, a large number of intraresidual distances (>100) can be measured from a single MTSL-labelled sample. In this study, six uniformly distributed MTSL positions provided a total of 2,288 distance restraints. The distance restraints were integrated into a structural model (Figure 8A), which shows tau in a much more compact form than previously expected from the EM images. However, the molecule is still loosely packed, highly flexible, and exchanges between a large number of conformations, consistent with large average values of the hydrodynamic radius (Figure 9A).

The C^α^ contact map, which reports on transient interactions that are found in many of the calculated structures, reveals an intricate network of long-range interactions in soluble tau (Figure 8B). In particular the hexapeptide in R3—a residue stretch that is essential for aggregation of tau into PHFs—is strongly involved into intramolecular contacts with both the C-terminal and N-terminal domain of tau. This includes a transient interaction with the amphiphatic C-terminal helix (Figures 7F and 8B). The second residue stretch with increased propensity for formation of an amphiphatic helix, ^114^Leu-Thr^123^, interacts with the N-terminal end of I1 as well as with repeats R1 and R2. Striking is also the compaction in the N-terminal 50 residues, in the region between I2 and P1, in P1 and P2, in repeats R1 and R2, and in repeats R3 and R4. In agreement with the paper clip model proposed by FRET measurements [27], the N terminus weakly interacts with the C terminus (Figures 7E, 7F, 8B, and S4). Interestingly, the proline rich region P2 has many contacts with distant areas of the sequence, such as R1, R2, R4, and the amphiphatic helix at the C terminus, suggesting that phosphorylation of residues in P2 may modify the ensemble of tau conformations, thereby promoting or delaying aggregation into PHFs. Moreover, the intramolecular interactions between its repeat and proline-rich regions might prime the tau protein for MT binding [45].

### Single-Residue Definition of the Hot Spots of the Tau-MT Interaction

The MAP tau is a critical regulator of diverse MT functions [9,45,52]. The repeat domain with its four repeats is essential for MT assembly, however, in the absence of the two flanking regions P2 and R', the repeat domain binds only weakly to MTs. The flanking domains, on the other hand, bind to MTs even in the absence of the repeats. This has led to the proposition of the “jaws” model of tau whereby the regions flanking the repeats are considered as targeting domains, responsible for positioning tau on the MT surface, and the repeats that act as catalytic domains for MT assembly [52,56].

**Figure 11:**
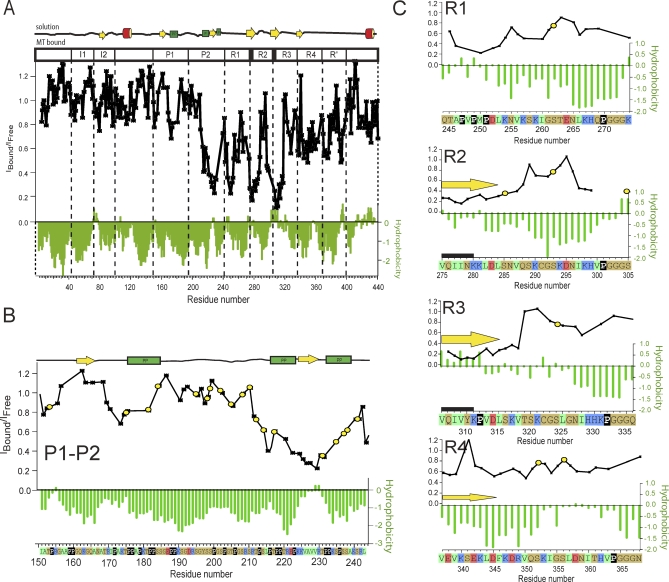
Comparison of MT-Binding Profile with Hydrophobicity Pattern of Tau (A) NMR signal intensity ratios between signals observed for htau40 in the MT-bound and in the free state (see also Figure 10B) are shown as stars and connected by lines. Hydrophobicity values calculated according to the Kyte-Doolittle scale are shown as green bars. Hydrophobic regions have positive or small negative hydrophobicity values. The location of transient secondary structure observed in unbound htau40 (i.e., as monomer in solution) is indicated as schematic on top. In (B) and (C) the regions that are important for interaction with MTs are shown in detail. The amino acid sequence is shown below. It is colour coded according to Figure 1B. The regions of tau that transiently populate β-structure and polyproline II helix in the unbound state are marked by yellow arrows and green bars. Phosphorylation sites are marked by yellow circles.

Here we probed the interaction of htau40 with MTs using solution-state NMR spectroscopy. Despite the fact that tau molecules are invisible to solution-state NMR when they are bound to MTs owing to the high molecular weight of the complex, information about the residues of tau that are important for binding to MTs can be obtained when the exchange between the fully bound form and the free state is sufficiently fast. In this case, the observed NMR signals will be an average of the resonances originating from the unbound and bound forms of tau, causing changes in NMR signal position and intensity. The strength of these changes will depend on the conformation and chemical environment in the bound state and the concentration of the bound species. Particularly striking was the pattern of NMR signal intensity ratios in the presence and absence of MTs (Figures 10B and 11A). Four highly localized regions were revealed, in which, because of chemical exchange between the unbound and MT-bound state, HN signal intensities were broadened below 40% of their value in the unbound state: ^225^KVAVVRT^231^, ^245^TAPVPMPDL^253^, ^275^VQIINKKLDLSNV^287^, and ^306^VQIVYKPVDLSKV^318^. In these regions, intensity minima were found for V228, M250, I278, and V309, respectively (Figure 11B and 11C). In agreement with the NMR data, deletion analysis mapped the MT-binding activity of the proline-rich region to residues K^224^-N^255^ and in particular to the stretch ^225^KVAVVRT^231^. Moreover, site-directed mutagenesis indicated that K^224^, K^225^, and R^230^ are important for MT-binding and -assembly [45]. It is noteworthy that the region ^225^KVAVVRT^231^ is conserved between tau and two other MAPs, MAP-2 and MAP-4 [45]. The importance of ^275^VQIINKKLDLSNV^287^ for MT-binding is supported by biochemical analyses that reported a strong reduction of MT binding affinity upon mutation of K^274^, K^280^, K^281^ to alanine [57]. Importantly, the hot spots of interaction are separated by residue stretches that show smaller chemical shift changes and less signal broadening (Figures 10 and 11). These residue stretches might act as flexible linker sequences and suggest that tau protein can assume multiple conformations on the surface of MTs. Moreover, the flexible structure may allow tau to be easily displaced from the MT lattice, consistent with the rapid diffusion of tau in neurons [58].

In addition to the hot spots of MT-interaction, signal broadening and chemical shift changes were observed for most residues in R4 and R' and extended to a weaker degree even up to the C terminus, indicative of transient interactions of these regions with MTs. However, in contrast to the proline-rich region P1 and repeats R1, R2, and R3, no clear minimum in NMR signal intensities was observed in repeat R4, indicating that R4 may not be very important to the interaction between tau and MTs. This observation is in agreement with biochemical studies that suggested a core-MT binding domain comprising the N-terminal side of the repeat region [59]. On the other hand, it is in contrast to the view that tau possesses multiple independent tubulin-binding sites [7]. As far as the projection domain is concerned, significant chemical shift changes were observed for residues in insert I2 and in the region with helical propensity (^114^Leu-Thr^123^), consistent with the finding that the projection domain regulates the spacing of MTs [60].

Little is known about the nature of the cognate tau binding sites in tubulin. Based on digestion experiments it is believed that tau binds to the acidic carboxyl tail of tubulin, which is supposed to be exposed on the surface of MTs [61]. Moreover, mutational analysis pointed to the importance of positively charged lysine residues (K^274^, K^280^, K^281^) in tau for MT-binding, suggesting that electrostatic interactions are important for the tau-MT binding [45]. On the other hand, the homologues region in R1 contains only a single positively charged residue (K^254^) at the edge of the most affected residue stretch, but does contain a negatively charged residue (D^252^). Similarly, the hot spots of MT-binding in R2 and R3 also contain a negative charge (Figures 1B and 11), suggesting that the tau-MT interaction might be more complex. Indeed, there is a striking correlation between the NMR-based MT-interaction profile and the hydrophobicity pattern of tau (Figure 11A). The 13-residue stretch in R3 (^306^VQIVYKPVDLSKV^318^), which shows the strongest chemical exchange contribution in the presence of MTs, is the most hydrophobic region of 441-residue tau (Figure 11C). Consistent with the importance of hydrophobic interactions, substitution of the tyrosine residue in this residue stretch by an asparagine (Y310->N) reduced the MT affinity of tau [59]. Maxima are also found in the hydrophobicity profile for the other three hot spots of MT-interaction (^225^KVAVVRT^231^, ^245^TAPVPMPDL^253^, ^275^VQIINKKLDLSNV^287^), whereas the homologous region in repeat R4 only has a few hydrophobic residues but has many charged residues, and is less affected by the presence of MTs (Figure 11C). Further support for the importance of hydrophobic interactions for formation of the tau-MT complex comes from the MT-binding site in P2: ^225^KVAVVRT^231^ is the most hydrophobic residue stretch in the proline-rich regions P1 and P2 (Figure 11B). Taken together, the NMR and biochemical data suggest a complex mechanism of tau-MT interaction involving both electrostatic and hydrophobic contacts.

Over 30 phosphorylation sites have been identified in tau, many of which are elevated in AD [62,63]. Prominent sites are located in the flanking domains, e.g., S^199^, S^202^, T^205^, T^212^, S^214^, T^231^, S^235^ before the repeats, S^396^, S^404^, S^422^, and others after the repeats (Figures 11B and 11C). The major sites within the repeats are located in the KXGS motifs, i.e., S^262^, S^293^, S^324^, and S^356^. These sites are phosphorylated by the kinase MARK, which results in the detachment of tau from MTs [64]. Interestingly, S^262^, S^293^, S^324^, and S^356^ are not part of the hot spots of MT-interaction, suggesting that phosphorylation at these sites might inhibit MT-binding by long-range electrostatic interactions. Alternatively, or in combination, phosphorylation can induce conformational changes that are incompatible with MT-binding. On the other hand, T^231^ is right in the middle of the MT-binding region in the proline-rich domain P2, making even a steric inhibition of MT-binding possible. Another potential mechanism is stabilization of a polyproline II helix by phosphorylation. Phosphorylation of residues within the fragments ^216^PTPPTREP^223^ and ^232^PPKSPSSA^239^ might stabilize their nascent polyproline II helical propensity, such that the resulting conformation is no longer able to efficiently bind to MTs.

Why would a natively unfolded protein evolve to stabilize axonal MTs? To consider this, we note that MTs bind a variety of proteins, some of which are natively unfolded (e.g., the tau-MAP2-MAP4 family), but others are typical well-folded molecules. Two cases in point are kinesin and doublecortin, both of which bind to the MT surface in a periodic and well-defined fashion. By contrast, the MAPs are rather diffusely distributed over the MT surface, there is little detectable periodicity, the MAPs bind relatively weakly, and they diffuse rapidly off MTs and along them [65]. To complicate matters further, binding of MAPs involves the C-terminal tails of tubulin subunits, which are themselves natively unfolded [61]. Not surprisingly, much of the N- and C-terminal domains of tau are highly flexible even when the repeat domain is attached to MTs [22]. Several explanations have been invoked to explain the functions of unfolded proteins such as MAPs [66]: They can act as entropic bristles to keep the spacing between MTs and other cell components (and indeed large MAPs keep larger spacings than small MAPs [60]); serve as assemblers for multisubunit structures (e.g., to pre-assemble tubulin into oligomers for incorporation into MT); serve as docking sites for enzymes (e.g., kinases and phosphatases for the case of MT-bound MAPs); and may even have regulatory functions for MT-related functions (e.g., interaction with motor proteins of axonal transport [67]). Thus, the multiplicity of functions would correspond to a multiplicity of conformations. Many of the above functions are inferred from biochemical evidence without detailed knowledge of the responsible residues and conformations of tau. The identification of residues reported here will provide a basis for future experiments to clarify the interactions of tau with interaction partners in cells, and hopefully the changes that occur during neurodegenerative tauopathies.

## Materials and Methods

### Disordered protein statistics.

Assignments of disordered proteins (IDPs) resolved by other groups with the corresponding protein size were found either in the BMRB databank (http://www.bmrb.wisc.edu/) or in publications listed in the PubMed (http://www.ncbi.nlm.nih.gov/pubmed/) databank.

### Assembly of MTs.

Porcine brain tubulin was purified and incubated at concentrations higher than 200 M in MT assembly buffer (100 mM Pipes, [pH 6.9], 1 mM EDTA, 1 mM MgSO_4_, 1 mM dithiothreitol) in the presence of 1 mM GTP at 37 °C for 5 min. After addition of 100 M Paclitaxel (Sigma-Aldrich) the polymerization was performed for 20 min at 37 °C. Analysis of MTs showed that MTs remained stable over the entire duration of the NMR experiments.

### Spin labelling of tau.

To label htau40 cysteine-containing mutants with the nitroxide spin label MTSL (Toronto Research Chemicals), DTT was removed before labelling from the buffer by using size exclusion chromatography (PD-10 columns, GE Healthcare), and the proteins were equilibrated in PBS buffer (pH 7.4). Free sulfhydryl groups were reacted with a 5-fold molar excess of the MTSL solubilized in ethyl acetate, at 21 °C for 2.5 h. Unreacted spin label was removed by using PD-10 columns equilibrated in 50 mM Na phosphate buffer (pH 6.8), and spin-labelled proteins were concentrated by using Amicon Ultra-15 (molecular weight cutoff, 3,000) (Millipore).

### NMR spectroscopy.

Protein concentrations were between 0.2–0.9 mM of htau40. NMR spectra were acquired on a Bruker Avance 900 spectrometer equipped with a cryogenic probe. Aggregation did not occur under these low temperature conditions. 3-D (HA)CANNH [30] (100 [F1] × 72 [F2] × 1 K [F3] complex data points) and HNN [31] (100 [F1] × 100 [F2] × 1 K [F3] complex data points) experiments (four scans, 1.2-s recovery delay, for each experiment roughly one day of measurement time) were collected. To enable and validate assignment of MT-bound htau40, a 3-D (HA)CANNH experiment was measured at 20 °C (total experiment time: ∼1.5 d). NMR data were processed and analyzed using NMRPipe [68] and Sparky 3 (T. D. Goddard and D. G. Kneller, http://www.cgl.ucsf.edu/home/sparky).

Secondary shift values were calculated as the differences between measured C^α^ chemical shifts and the empirical random coil value for the appropriate amino acid type [69]. Random coil values for histidines, glutamates, and aspartates were taken from Wishart and Sykes [70], as the chemical shifts of these residues are particularly sensitive to pH. To estimate the secondary structure propensity in contiguous segments of htau40, the observed C^α^ chemical shifts were normalized by the empirically determined secondary shift expected for that residue type in a regular secondary structure (β-sheet or α-helix) conformation [70], summed and normalized by the number of residues in the segment.

^3^J(H^N^H^α^) scalar couplings were measured using an intensity modulated HSQC [71] on a Bruker 900 Avance spectrometer (32 scans, relaxation delay 1.2 ms, 2τ = time for evolution of ^3^J_HNHα_: 18 ms). Coupling values were calculated from the intensity ratios using the relation *S*_cross_/*S*_diag_ = cos(π^3^J_HNHα_2τ). Secondary ^3^J(H^N^H^α^) scalar couplings were calculated as the difference between experimental ^3^J(H^N^H^α^) scalar couplings and random coil values [72].

^15^N R_1ρ_ relaxation rates were measured at 5 °C on a Bruker Avance 700-MHz spectrometer using a spinlock frequency of 2 kHz and relaxation periods of 20, 100, 220, and 300 ms. Relaxation times were calculated by fitting an exponential function to the decaying signal integrals.

One-bond N-H RDCs (D_NH_) were determined by using an inphase-antiphase (IPAP)-HSQC [73]. D_NH_ values were calculated as the difference between splittings measured in the isotropic phase and in a sample, in which htau40 had been aligned in 5 mg/ml Pf1 bacteriophage (Asla). Errors estimated on the basis of the signal-to-noise ratio are 0.2 Hz for D(_NH_) and 0.4 Hz for ^3^J(H^N^H^α^) couplings, respectively.

### NMR diffusion measurements.

For determining the hydrodynamic radius, htau40 was dissolved in 99.9 % D_2_O, 50 mM phosphate buffer (pH 6.9). The samples contained dioxane (concentration ∼2%) as an internal radius standard and viscosity probe [43]. 1-D ^1^H spectra were collected employing the standard Bruker pulse program ledbpgp2s. The gradient strength was linearly increased from 2% to 95% of the maximum gradient strength in 16 steps, with 100% gradient strength corresponding to 56.9 G/cm. For each ^1^H spectrum 128 scans and 16 K complex with a spectral width of 7,200 Hz were acquired. Signal intensities corresponding to the aliphatic region of the ^1^H spectra (3.3–0.5 ppm) were readout with the TOPSPIN T_1_/T_2_ Relaxation module (Bruker Instruments). The diffusion data (signal intensity versus gradient strength) were fitted to exponential functions using Igor Pro 5.01 (WaveMetrics). From the apparent diffusion coefficients of htau40 and dioxane and the known Stokes radius of dioxane (2.12 Å), Stokes radii of monomeric htau40 were calculated [74].

### Measurement of PRE.

PRE broadening was investigated using ^15^N-labelled htau40 at a concentration of 15 μM (MTSL at A15C, A239C, and C291 + C322) and 50 μM (MTSL at A416C, A384C, and A72C) in 50 mM phosphate buffer at (pH 6.8). PRE effects were measured from the peak intensity ratios between two 2D ^15^N-^1^H HSQC NMR spectra acquired in the presence of the nitroxide radical and after addition of 4 mM DTT (heated to 45 °C for 15 min before measurement) to the same sample. Addition of DTT will cleave the MTSL tag from the cysteine residue, such that the spin label is no longer attached to the protein and the protein is in the diamagnetic state. Oxidation of the MTSL tag with ascorbic acid, gave very similar results (Figure S3B).

To exclude intermolecular contacts as cause for PRE line-broadening, a mixture of 15 μM ^15^N-labelled htau40-(C291A/C322A) mutant (without any cysteine residues) and 15 μM ^14^N-labelled htau40-A239C-MTSL was measured at 5 °C (Figure S3A). In addition, intramolecular PRE broadening at htau40 concentrations of 15 and 50 μM were compared (Figure S3C).

### Calculation of distance restraints.

Distance restraints were calculated as described from the intensity ratio between two 2D^ 15^N-^1^H HSQC NMR spectra, in the diamagnetic and paramagnetic states of the protein [39]. To reduce the impact of peak overlap, for each residue the average of its own intensity ratio *I*_para_ */I*_dia_ and that of the preceding and following residue was calculated. These smoothed intensity ratios were linearly fit for the enhancement of the transverse relaxation rate by the unpaired electron [75]. For calculation of distance restraints, amide proton *R*_2_ values were approximated by experimental amide nitrogen *R*_1ρ_ values [76]. The correlation time for the electron-nuclear interaction was set to 4 ns, in agreement with previous studies [39]. For peaks broadened beyond detection, distances were set to 7 ± 5 Å. Peaks with intensity ratios below 0.95 were restrained to the calculated distance ±5 Å by using a harmonic square well potential. For residues that were not broadened in the paramagnetic state, a lower distance bound of 25 Å was used. All distances were imposed as restraints between the C^α^ atom of the residue with the cysteine-MTSL group and residue-specific amide protons.

### Structure calculation and analysis.

Structure calculations were performed using XPLOR-NIH, version 2.9.7 [40]. An all-atom representation of htau40 was used. Structural energy terms from steric repulsion, bond length, bond angles, dihedral angles, and favoured regions of the Ramachandran map were employed.

For restraining a single molecule simultaneously by all PRE distance restraints, torsion angle dynamics were started at 3,000 K with the temperature reduced to 20 K, followed by a short energy minimization. 50 structures were calculated starting from a random coil. The seven lowest-energy structures that satisfied the 2,288 distance restraints with no violations greater than 1 Å were used for calculation of the average contact map shown in Figure 8B.

Single molecule calculations were followed by ensemble calculations, in which distance restraints do not have to be fulfilled by a single molecule, but collectively by the ensemble of molecules. Ensemble calculations were started from the lowest-energy structure obtained in the single molecule calculation (see above) and performed in two rounds. Initially, distance restraints were enforced onto an ensemble of 30 molecules [41]. Torsion angle dynamics were used with the temperature reduced from 10,000 K to 5,000 K. The lowest energy structure obtained from this first round of ensemble averaging was subjected to another round of structure calculation using an ensemble size of 5. Torsion angle dynamics was used and the temperature was reduced from 3,000 K to 1,000 K. A total of 100 structures were calculated. The average contact map obtained from the seven lowest-energy structures of the ensemble was very similar to the one obtained from single molecule calculations (Figure S4).

## Supporting Information

Figure S1 Degeneracy of C^α^ Connectivity (A) Residue Y18 points back to T17, but for 18 other residues the C^α^ frequency also lies within a range of +/− 0.2 ppm (red strip) of the C^α^(i-1)peak of Y18.
(B) Comparison of C^α^ chemical shift degeneracy observed in htau40 (441 residues) with values reported for the globular proteins calmodulin (148 residues), N-terminal domain enzyme I (259 residues), maltose-binding protein (370 residues), and malate synthase G (723 residues).
(411 KB PDF)

Figure S2 Robustness of Residual Secondary Structure of htau40 in Solution (A),(C),(D), Comparison of C^α^ secondary chemical shifts, ΔC^α^, observed in htau40 and the tau fragments K32, K25, and K10, respectively (all at 5 °C).
(B) Comparison of C^α^ secondary chemical shifts, ΔC^α^, observed in htau40 at 278K and 293K. Red lines indicate *y* = *x*.
(337 KB PDF)

Figure S3 Control PRE Measurements (A) Intensity ratio (*I*_param_/*I*_diam_) obtained from HSQC spectra recorded on 15 μM ^15^N-labelled htau40-(C291A/C322A) (i.e., htau40 without any cysteine residues) in the presence of 15 μM ^14^N-labelled htau40-A239C-MTSL in the paramagnetic and diamagnetic state. No significant deviations from unity were observed, indicating that decreases in intensity ratios are not due to aggregation, and can be attributed instead to intramolecular long-range contacts in native htau40.
(B) Comparison of PRE intensity ratios for different diamagnetic reference states. For the data shown by blue bars, DTT was added to the sample, whereas for the red line ascorbic acid was used to quench the spin label.
(C) Comparison of intensity ratios obtained from HSQC spectra recorded on 15 μM (red line) and 50 μM (blue bars) ^15^N-labelled htau40-A239C-MTSL in the presence and absence of DTT.
(202 KB PDF)

Figure S4 Average Contact Map for the Seven Lowest-Energy Structures Obtained from Ensemble Calculations with an Ensemble Size of 5 (see Materials and Methods) A continuous grey scale from 3 Å (black) to 22 Å (white) is used.
(2.14 MB PDF)

## Abbreviations

AD: Alzheimer disease
FRET: Förster resonance energy transfer
HSQC: heteronuclear single quantum coherence
htau40: longest tau isoform found in the human central nervous system
MAP: microtubule-associated protein
MT: microtubule
MTSL: (1-oxy-2,2,5,5-tetramethyl-D-pyrroline-3-methyl)-methanethiosulfonate
NMR: nuclear magnetic resonance
PHF: paired helical filament
PRE: paramagnetic relaxation enhancement
RDC: residual dipolar coupling
SAXS: small angle x-ray scattering
