# Supplementary material for: Structural Polymorphism of 441-Residue Tau at Single Residue Resolution
Source: PLoS Biol. 2009 Feb 17;7(2):e1000034. doi: 10.1371/journal.pbio.1000034 (PMC2642882; doi:10.1371/journal.pbio.1000034)
Supplement: Supplementary file 1 [file pbio.1000034.sg001.pdf]

a

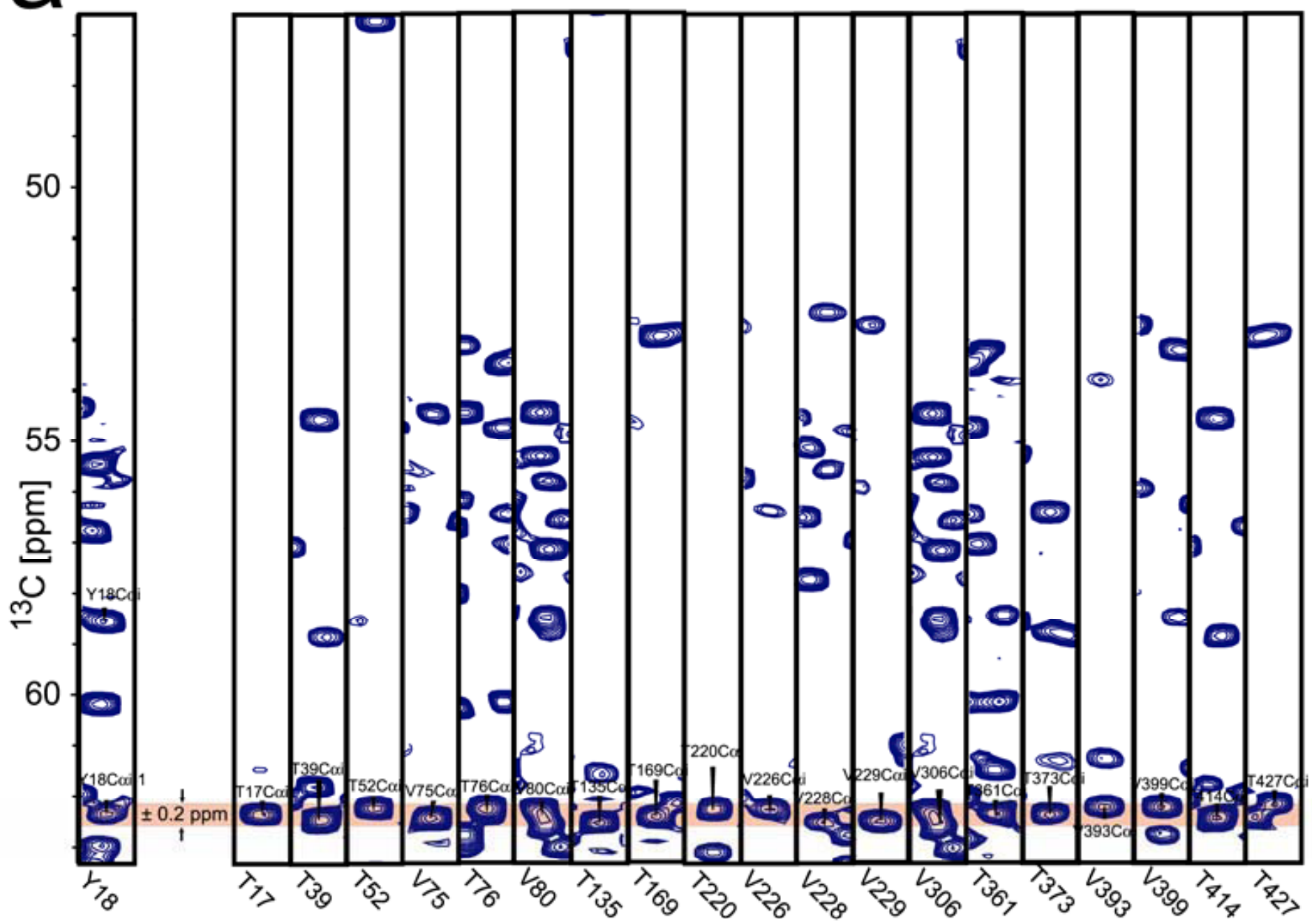

50
55
60
$^{13}$C [ppm]
± 0.2 ppm
Y18
T17
T39
T52
V75
T76
V80
T135
T169
T220
V226
V228
V229
V306
T361
T373
V393
V399
T414
T427


b

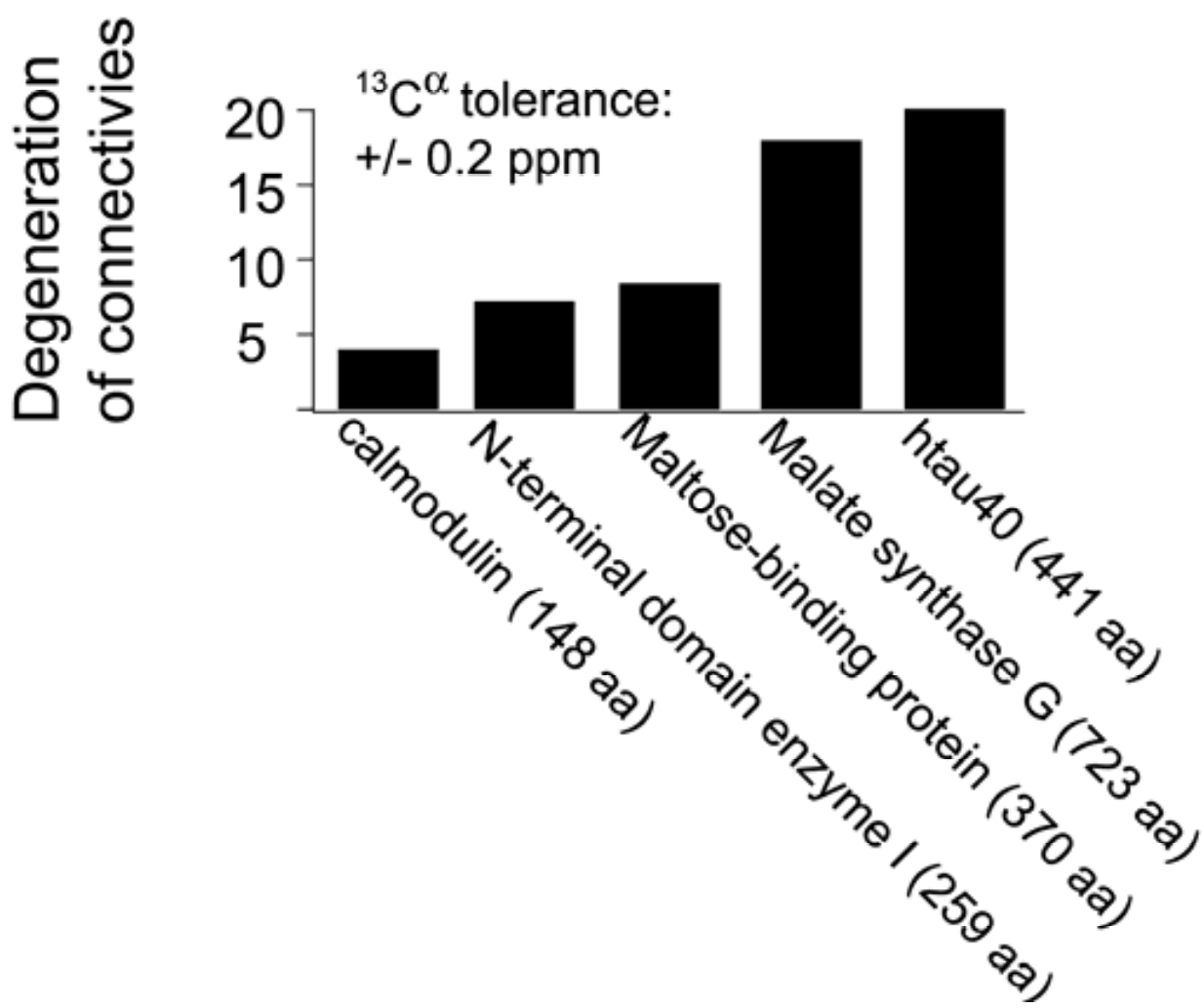

$^{13}$C$^{\alpha}$ tolerance:
+/- 0.2 ppm
Degeneration of connectivities
20
15
10
5
calmodulin (148 aa)
N-terminal domain enzyme I (259 aa)
Maltose-binding protein (370 aa)
Malate synthase G (723 aa)
htau40 (441 aa)
