# Supplementary material for: Structural Polymorphism of 441-Residue Tau at Single Residue Resolution
Source: PLoS Biol. 2009 Feb 17;7(2):e1000034. doi: 10.1371/journal.pbio.1000034 (PMC2642882; doi:10.1371/journal.pbio.1000034)
Supplement: Supplementary file 2 [file pbio.1000034.sg002.pdf]

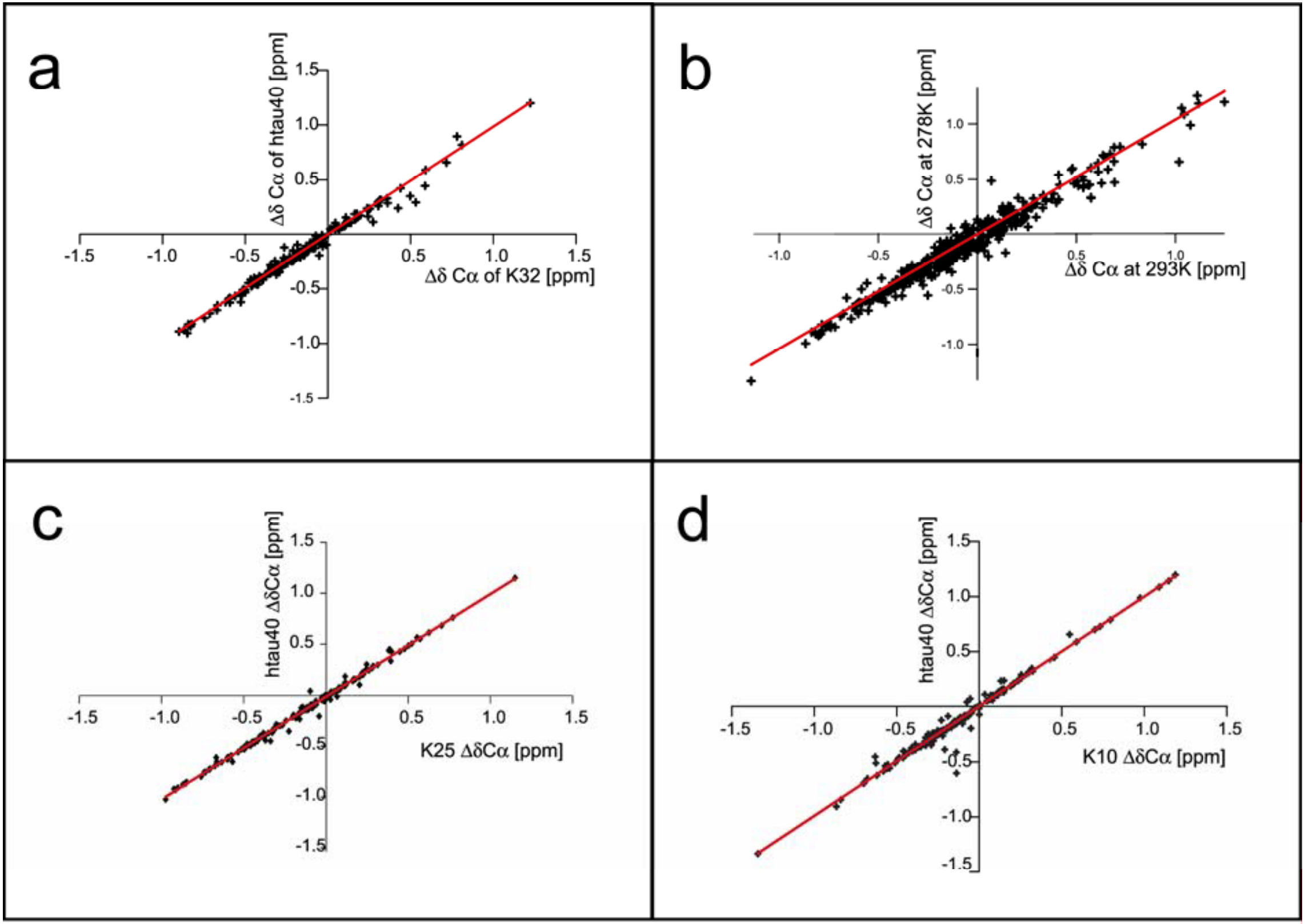
a
Δδ Cα of htau40 [ppm]
Δδ Cα of K32 [ppm]
b
Δδ Cα at 278K [ppm]
Δδ Cα at 293K [ppm]
c
htau40 ΔδCα [ppm]
K25 ΔδCα [ppm]
d
htau40 ΔδCα [ppm]
K10 ΔδCα [ppm]
