# Supplementary material for: Structural Polymorphism of 441-Residue Tau at Single Residue Resolution
Source: PLoS Biol. 2009 Feb 17;7(2):e1000034. doi: 10.1371/journal.pbio.1000034 (PMC2642882; doi:10.1371/journal.pbio.1000034)
Supplement: Supplementary file 3 [file pbio.1000034.sg003.pdf]

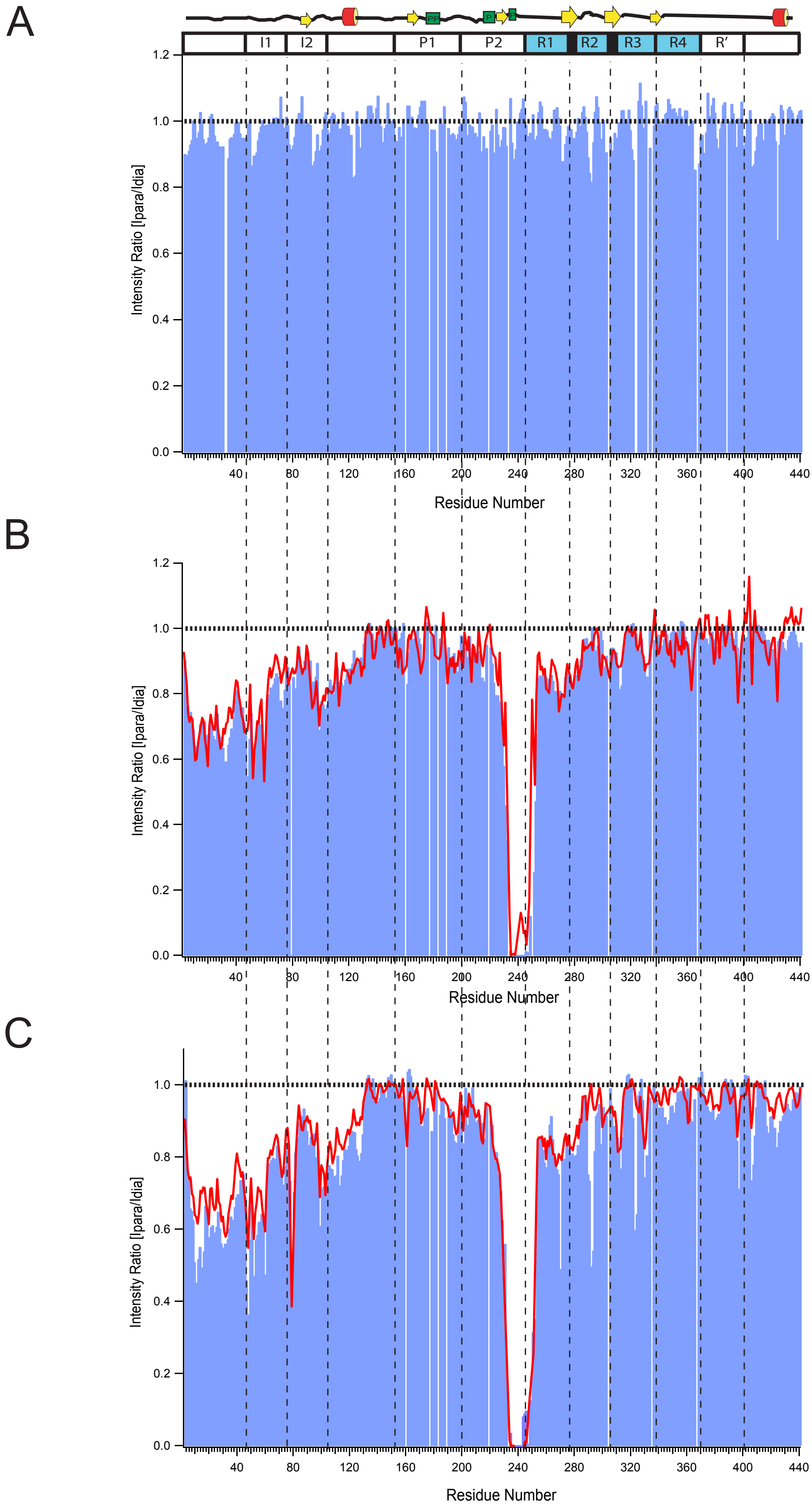
A
I1
I2
P1
P2
R1
R2
R3
R4
R'
Intensity Ratio [Ipara/Idia]
Residue Number
B
C
