# Supplementary material for: Structural Polymorphism of 441-Residue Tau at Single Residue Resolution
Source: PLoS Biol. 2009 Feb 17;7(2):e1000034. doi: 10.1371/journal.pbio.1000034 (PMC2642882; doi:10.1371/journal.pbio.1000034)
Supplement: Supplementary file 4 [file pbio.1000034.sg004.pdf]

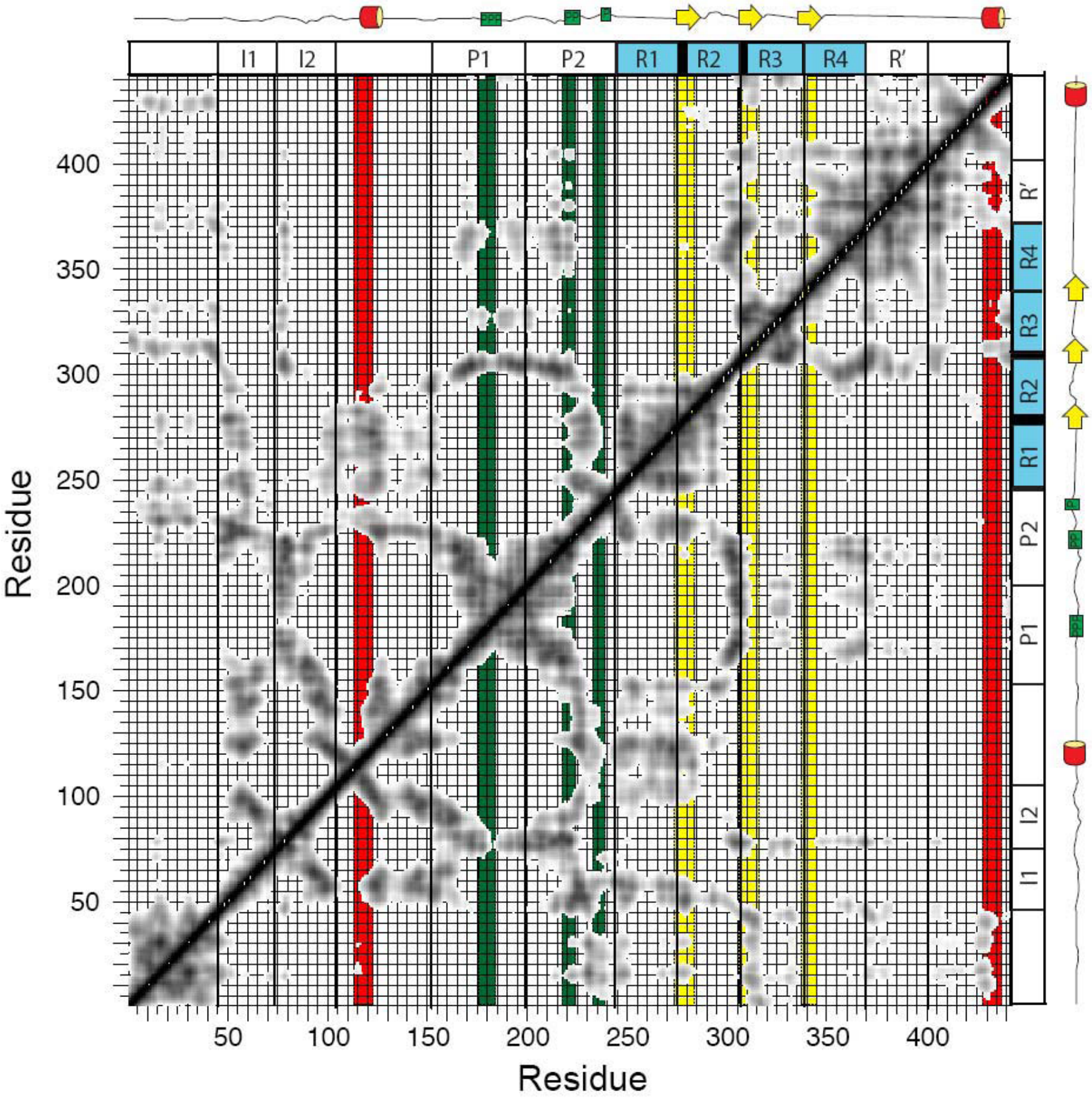

I1
I2
P1
P2
R1
R2
R3
R4
R′
Residue
50
100
150
200
250
300
350
400
Residue
